# Impact of drying methods on the yield and chemistry of *Origanum vulgare* L. essential oil

**DOI:** 10.1038/s41598-022-07841-w

**Published:** 2022-03-09

**Authors:** Lucia Caputo, Giuseppe Amato, Pietro de Bartolomeis, Laura De Martino, Francesco Manna, Filomena Nazzaro, Vincenzo De Feo, Anna Angela Barba

**Affiliations:** 1 Dipartimento di Farmacia, Università degli Studi di Salerno, via Giovanni Paolo II 132, 84084 Fisciano, Salerno Italy; 2 Istituto di Scienze dell’Alimentazione, CNR, Via Roma 60, 83100 Avellino, Italy; 3 Caselle Società Agricola Srl, Via Mare Mediterraneo 18, 84098 Pontecagnano, Salerno Italy

**Keywords:** Plant sciences, Natural products

## Abstract

Oregano (*Origanum vulgare* L.) is mainly cultivated, both as fresh and dried herb, for several purposes, such as ailments, drugs, and spices. To evaluate the influence of some drying methods on the chemical composition of the essential oil of oregano, its aerial parts were dehydrated by convective drying techniques (shade, static oven), microwave-assisted heating (three different treatments) and osmotic treatment. The oils were analyzed by GC-FID and GC–MS. The highest essential oil yield was achieved from microwave and shade drying methods. In total, 39 components were found, with carvacrol (ranging from 56.2 to 81.4%) being the main constituent; other compounds present in lower amounts were *p*-cymene (1.6–17.7%), γ-terpinene (0.8–14.2%), α-pinene (0.1–2.1%), thymol methyl ether (0.4–1.8%) and thimoquinone (0.5–3.5%). The essential oil yields varied among the different treatments as well as the relative compositions. The percentages of *p*-cymene, γ-terpinene and α-pinene decreased significantly in the dried sample compared with the fresh sample; on the other hand, carvacrol, isoborneol and linalool increased significantly in the dried materials. The choice of the drying method for obtaining the essential oil therefore appears crucial not only in relation to the higher yield but also and above all in reference to the percentage presence of components that can direct the essential oil toward an appropriate use.

## Introduction

In the Mediterranean area, *Origanum* genus (Lamiaceae) comprises 39 amply distributed species, among which the perennial herbaceous plant *O. vulgare* L., commonly known as “oregano”^1^. The plant is rich in phenolic compounds with high antioxidant and antibacterial properties. Even if the species is indigenous to Mediterranean regions, such as Italy, Greece and Spain, it is also present all over the world throughout the year. However, fresh plants have limited use; in contrast, the dry form of the spice is frequently used in the food industry.

Essential oils (EOs) are natural complex mixtures of volatile compounds usually obtained by hydrodistillation and characterized by a strong odor. They can be synthesized by all plant organs and possess various biological activities, such as antibacterial, antiviral, antifungal and insecticide activities*.* Currently, they maintain attractive characteristics for medical, cosmeceutical and nutraceutical formulations due to their natural origins and safe and economic features.

The extractive yield, chemistry and related bioactivities of EOs are highly affected by exogenous factors such as soil features, harvest time, plant development stage, geolocation and extraction and/or drying methods^2^.

Aromatic plants, such as basil, oregano, and sage, are normally used as dried spices. Drying is based on simultaneous heat and mass transfer phenomena and is widely applied to preserve properties, such as aroma, taste and nutritional factors, and to reduce bacterial growth, achieving a longer shelf life^3^.

There are literature studies in which different methods were applied for oregano drying. In particular, innovative protocols, also applied as combined techniques (microwave drying, microwave pulsed heating, preconvective ventilation, use of vacuum), are currently being investigated^4–6^.

The yield and chemical composition of EOs could change depending on the drying method^3^: in fact, enzymatic and non-enzymatic reactions during the drying of fresh plants could modify the phytochemical composition, which can have repercussions on their final quality^7^. It has been proven that the presence or absence of a single constituent can cause important changes in the biological activities of aromatic oils^8^. A high content of bioactive compounds represents desired characteristics for medically dried herbs, while for food use, such as in the case of culinary dried herbs, the quality is represented by the colour and a fresh-like characteristic aroma^9^. For this reason, the selection of the drying method appears crucial^10^.

There are several drying methods that can be used. Traditional or conventional treatments are based, prevalently, on convective transport phenomena with heat and mass transfer. In some industrial applications, the principles underlying osmotic processes can be applied to the dehydration of plant matrices. Motivations such as improved product quality and reduction in manufacturing costs (energy and other kinds of resources) are driving research by both academia and industry to develop nonconventional methods.

In traditional heating processes, energy is transferred to material by convection, conduction and radiation phenomena due to thermal gradients and through the external surface of materials. Solar drying is the oldest drying method based on radiative (heat transfer) and convective (mass transfer) phenomena and is still used in several tropical or subtropical countries^11^. During this process, fresh herbs are exposed directly to sunlight, which often determines considerable colour and aroma loss in dried herbs^12^. Shade drying methods always utilize solar energy as a heating source, preserve light-sensitive substances and reduce oxidation processes. However, the shade drying time is longer than sun drying, but due to its low cost, the shade drying method is still widespread in rural regions or in small businesses^12^. The use of cabined (or ovens) and bed-type dryers (tunnel, tray, rotary conveyor and so on) constitutes the industrial response to enhance and standardize drying convective operations. These kinds of driers are mostly suitable for solid materials such as chunked products, vegetables and herbs, and fruit grains^13^, allowing various process conditions such as exercise drying temperature and drying time and velocity^14^.

The stabilization of food products by osmotic dehydration is achieved by immersion in hypertonic solutions (i.e., sugar, salt, other) prepared in dedicated tanks^13^. Water diffusion from tissues into the solution across cell membranes is driven by the higher osmotic pressure of the hypertonic solution. The rate of water diffusion mainly changes on the basis of factors such as the temperature and concentration of the osmotic solution and material size.

Microwave (MW) and radio frequency (RF) heating represent the latest advanced technologies in dehydration food processes. In particular, microwaves are electromagnetic radiations characterized by frequencies from 300 × 10^9^ Hz to 300 × 10^6^ Hz, on the basis of international agreements; frequencies of 915 MHz and 2450 MHz are dedicated to applications for industrial, scientific and medical scopes^15^. Microwave energy is due to direct interactions between the applied field and materials (loss mechanisms), interactions that cause the conversion of electromagnetic energy into thermal energy^16^. When intense loss mechanisms occur, high aliquots of energy conversion are expected. In particular, the high heating rate is a peculiar feature of microwave heating because it allows short treatment times (seconds or minutes) instead of hours if heating is done by conventional heating. This is due to the slow heat delivery rate from the material surface to the inner parts, as determined by the difference in temperature from a warm outside to a cool inside (fruits and vegetables have poor conductive thermal properties). In contrast, microwave energy can produce, under some conditions, volumetric heating and high-quality and bioactive compound preservation of treated vegetables^17^. From an energy-saving point of view, heating assisted by microwaves can be considered an intensified operation^18^, which constitutes one of the main reasons for growing industrial interest. Key factors in microwave heating are the dielectric properties of irradiated materials, i.e., capability of substances to interact with electromagnetic fields. On the basis of dielectric properties, dedicated microwave apparatuses, also named applicators, can be used in heating operations, and optimized protocols can be developed (definition of power, time, load configuration, etc.) can be used^16^.

The procedures used for drying material plants can influence the essential oil quantity and quality of aromatic species^19^. In fact, there are some previous papers about the effect of drying on the yield and the chemical composition of essential oils distilled from Lamaiceae, such as *Ocimum basilicum* L., *Thymus daenensis* Celak, *Mentha longifolia* L.^20,21^, but to our knowledge, studies on the changes in the quantity and quality of essential oil of *Origanum* genus depending on drying methods are scarce in the literature. In Calín-Sánchez et al.^4^, the influence of the drying method on the aroma quality of *Origanum majorana* species was evaluated applying three different dehydration methods: convective drying, microwave vacuum drying and a combination of convective pre-drying and vacuum-microwave finish-drying. The total concentration of plant compounds was reduced by most of the drying treatments performed, with the exception of microwave vacuum drying (240 and 360 W). In particular, the 240 W power protocol proved to be the best option for dehydrating oregano followed by the combined convection-microwave technique (50 °C, 240 W or 360 W). The two techniques assisted by microwave showed short drying times and a good quality of total concentration of volatile substances and excellent results in sensory evaluation^4^. In Soysal et al.^6^, three applied techniques for dehydration of oregano were compared: the combined intermittent convective-microwave technique, the continuous convective-microwave technique and the conventional convective one. It has been shown that both microwave-assisted techniques increase the drying speed of oregano compared to the convective one, which also presented the greatest energy consumption. In particular, the intermittent convective-microwave technique gave better results in terms of composition of the essential oil, which was found to be very similar to the profile obtained by convective drying at 50 °C, allowing to reach a good compromise between product quality dehydrated and speed and economy of the process^6^. Jałoszyński et al.^22^ studied the effect of oregano dehydration on the nutritional activity of the dried plant using three different techniques: freeze drying (− 60 °C, 65 Pa), convective drying (50, 60 and 70 °C) and vacuum oven (4–6 kPa, 240, 360 and 480 W). In relation to the antioxidant activity, freeze-drying proved to be the least destructive drying method but is the most laborious to apply. Moreover, it was observed that a higher phenolic content, in the dried oregano, can be achieved by reducing the temperature in the convection technique or by increasing the microwave power in the vacuum method anyway^22^.

These literature researches are examples of combined drying methods that can allow to achieve dried products with high quality but require expensive apparatuses and appropriate skills. More simple techniques, easy to apply, are thus desirable.

In this study, the effects of several drying techniques on the yield and chemical profile of aromatic oils obtained from *O. vulgare* L were evaluated. In particular, this research investigates the feasibility of applying simplified protocols, only based on microwave irradiation (not combined techniques), to achieve dried oregano products with good quality: the  features of the obtained essential oils and reduction of tissue damage, thanks to simplified processes characterized by fast drying kinetics with the final aim of improving production yields and production costs, will let to transferg laboratory procedures on a productive industrial scale in an easy manner.

## Results and discussion

### Dried products features

The residual moisture content was monitored during the drying treatments. Low moisture content values are mandatory to achieve stable matrices because they allow the inhibition or limitation of microbiological alteration of treated products. Literature studies reported a final moisture content of < 10% (wet basis) (this can be considered an average value due to the variability in the kind of herb—www.esa-spices.org) after drying treatments^23,24^. Moreover, the drying process could constitute an intermediate treatment to enhance or facilitate other operations, such as extraction or mechanical comminution. In this study, the plant samples were subjected to different drying treatments to determine their effect on essential oil chemical composition. In particular, the end points of the applied dehydration treatments were different due to the intrinsic potentiality or setting possibility of the applied drying method. Fresh oregano had a moisture content of approximately 70% (w/w). This value is lower than those reported in the literature^3–5^; a different moisture content in fresh products can be affected by natural (variety, ripening) and artificial factors (harvesting, storage, preparation methods, etc.). Shade (CONV1)- and hot air (CONV2)-dried batches showed residual humidity values of 37 and 5.3% (w/w) after 5 days at room temperature and 24 h at 50 °C, respectively. In oregano batches treated by microwave heating at 2300 W—10 min (MW1), 1150 W—15 min (MW2), and 460 W—35 min (MW3), the water contents were 8.6, 7.5 and 12.3%, respectively. Short treatment times (minutes not hours) are related to the ability of the fresh product, rich in water, to interact with microwaves^16,25^.

Osmotic treatment is a process that involves the partial removal of water from fresh fruits and vegetables to keep them longer. This treatment works by soaking food in close contact with higher osmotic pressure, sometimes referred to as a hypertonic or concentrated solution. The water then passes through the food in concentrated solution under the influence of the osmotic pressure gradient. It involves the lowering of activity water (aw) to microbiological safety conditions (< 0.8). In our experiments, the residual moisture content after the osmotic treatment was 48.5%.

**Figure 1:**
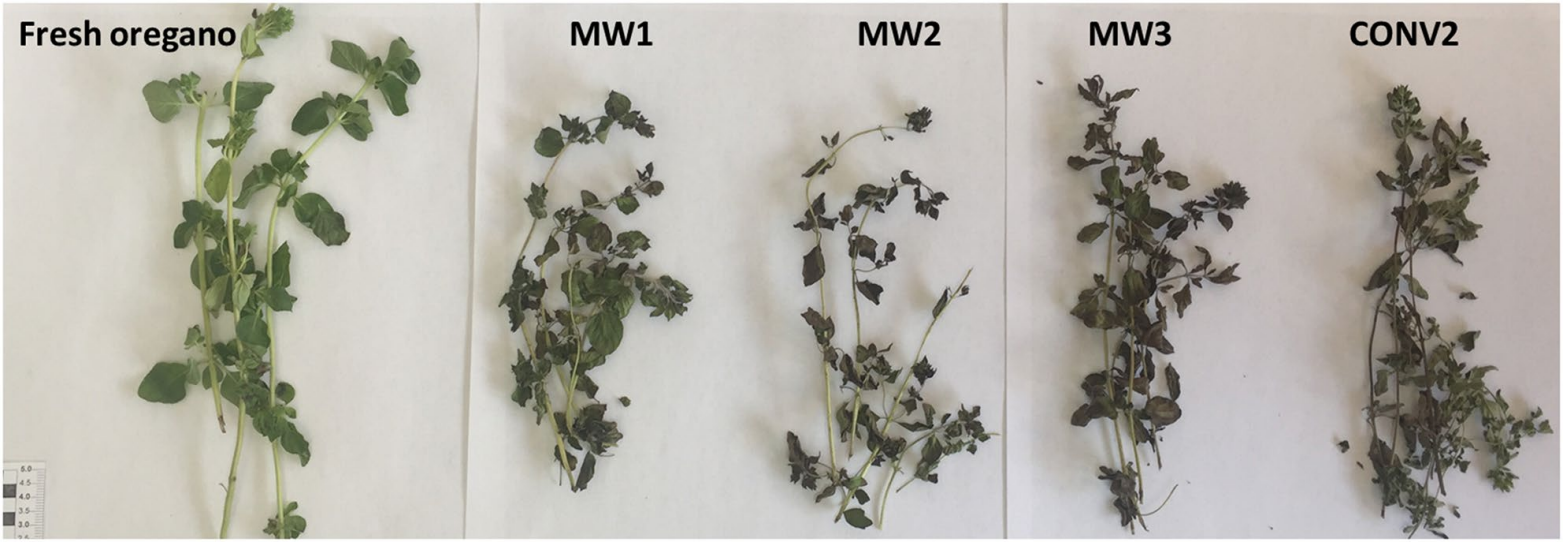
Oregano samples: fresh sample and dried samples.

A post drying visual examination showed browning effects on all dried products (on foliage, inflorescences, and twigs) and a certain degree of shrinkage (especially for leaves). Browning effects, however, are more evident in hot air-treated samples (CONV2) (Fig. 1). The observed differences are related to the drying physics aspects. In microwave-assisted drying processes, samples are subjected to less thermal stress; in fact, even if they are exposed to higher temperatures, the exposure time is considerably shorter. In this study, the maximum temperature values reached on oregano surface structures, measured during microwave irradiation, were in the range of 55–70 °C (hot spot values). Reduced thermal effects allow lower chemical degradation processes (losses of aromas, vitamins, proteins, color), preserving the sensorial and nutritional aspects^26^. On the other hand, the ultrarapid transport of matter (migration of humidity from herbs to the process environment) can cause severe tissue damage. The high speed of transport of matter can have repercussions on the structural properties (cracking events) compromising, even deeply, any subsequent processes such as rehydration and cooking of dried vegetables^16^.

It is therefore essential to choose the correct dehydration method according to the use of the plant. In fact, in sauces, spices with a bright color and not oxidized are preferred; this guarantees a greater presence in percentage of compounds with a strong biological action, useful for the pharmaceutical and nutraceutical sectors.

One method for assessing tissue damage in herbs and other plant matrices is the assessment of electrolyte loss, which constitutes an indirect measure of cell wall integrity^27–29^.

Tissue alterations of vegetables (and fruits), due to the injuries of thermal or other kinds of treatments or unsuitable storage conditions, result in an increase in the rate of ions released by the vegetal structures when placed in a dissolution medium^26^. In this research, to evaluate the effect of drying methods based on heat transfer (convective and radiative mechanisms) on tissue structure preservations of oregano dried samples, mineral leaching in distilled water was investigated.

**Figure 2:**
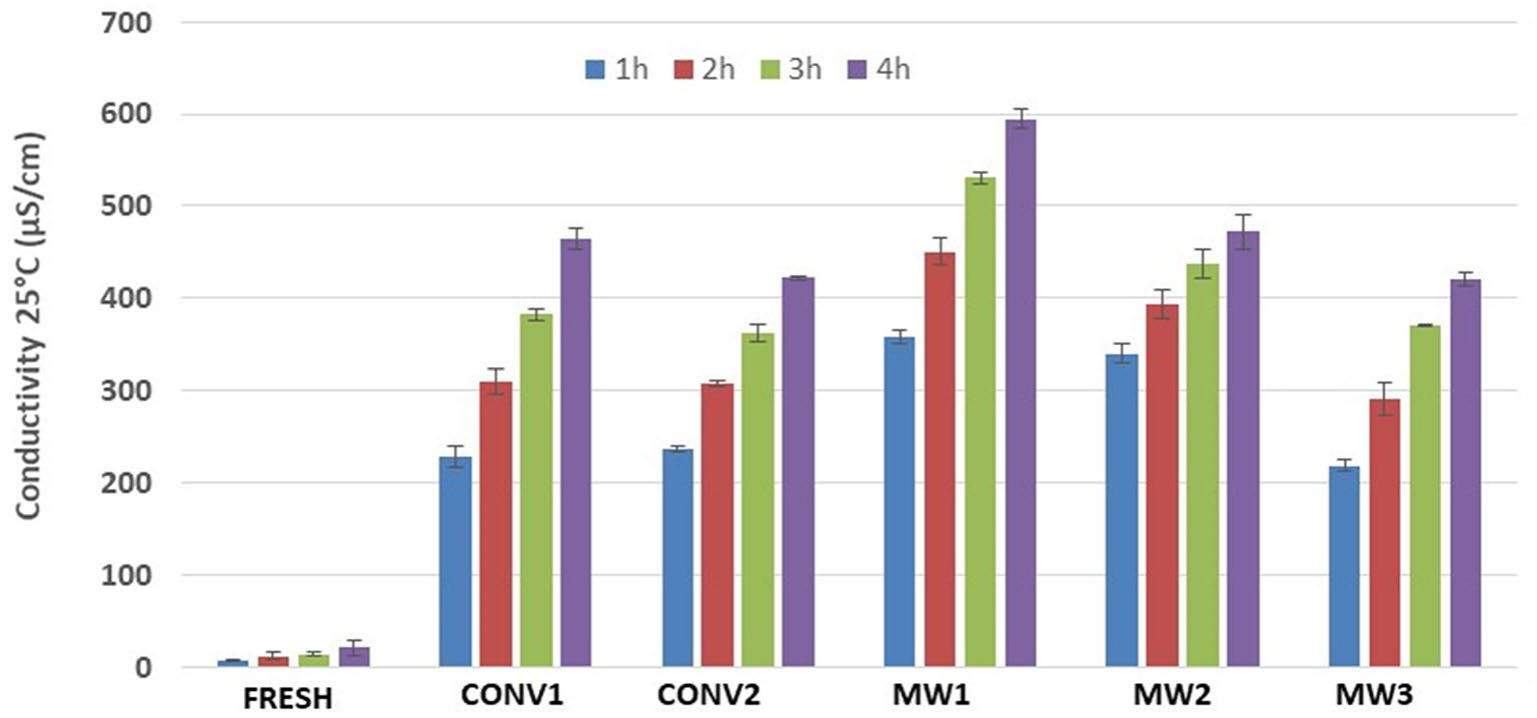
Electrolyte leakage from oregano samples (fresh and dried).

In Fig. 2, the results of electrical conductivity measurements are summarized. As shown, mineral losses increase over time for all the treated samples. Lower electrolyte leakage was found for MW3 dried products, in which best tissue preservation can be assumed. More disruptive characteristics were indeed shown by the MW1 protocol, as seen from the values of the aqueous bulk conductivity. After 48 h, the values of electrolyte leakage for the MW3 and MW1 dried products continued to show differences (694 ± 5 and 946 ± 8 µS/cm, respectively, data not shown), demonstrating an effective impact of microwave heating on vegetal tissue.

Shade (CONV1)- and hot air (CONV2)-dried samples exhibited comparable electrolyte loss values, confirming tissue damage due to dehydration via the CONV1 method, with damage mainly due to natural metabolic effects postharvest at room conditions, and via the CONV2 method, with damage mainly due to prolonged exposure at high temperature (after 24 h, 759 ± 28 and 725 ± 12 µS/cm, respectively, data not shown).

MW2 dried products present similar trends of samples MW1 and MW3 with intermediate conductivity values, which may suggest an in-between tissue stress.

### Impact of drying methods on the essential oil yield

Plants dried before distillation can usually have an increase or a reduction in yield^30^. These differences in values might be due to the drying time and the temperatures used in the different drying methods^31^. In particular, a higher yield was obtained from the samples dried with the MW3 and CONV1 methods (2.20% and 0.72% w/w, respectively). The yields of the volatile oils distilled from the fresh plant and the CONV2, MW1, MW2 and OT drying methods were 0.2, 0.4, 0.1, 0.2, and 0.0%, respectively. It has been reported that the volatile oil content is affected by the drying method, temperature, drying time, and plant species because the moisture level between samples dried with different methods could be highly changeable. For example, in *Thymus daenensis* Celak. the highest essential oil quantity was registered after drying, at 35 °C, in an oven^32^; instead, *O. vulgare* L. ssp. *hirtum,* after air-drying showed a minor change in the essential oil yield in comparison with the fresh plant material with respect to our data^33^.

### Effect of drying methods on essential oil composition

Table 1 reports the variation in *O*. *vulgare* essential oils obtained under the six different drying methods compared to the essential oil obtained from fresh plants. Altogether, 39 constituents were identified, accounting for 97.8–98.8% of the total oils. The highest number of constituents (36) was detected in the EO obtained after the MW2 drying treatment, while in the EO obtained from the fresh sample, only 15 components were found. These changes were probably due to the formation of new compounds by oxidation, glycoside hydrolysis, esterification, and/or other processes^34^.

**Table 1:** Effects of different drying methods on the essential oil composition of *Origanum vulgare*. Results are expressed as mean percentage ± standard deviation of three independent determinations.

Rt	Compound	Fresh	CONV1	CONV2	MW1	MW2	MW3	OT	KI^a^	KI^b^	Ident.^c^
12.548	α-Pinene	**2.1 ± 0.1**	1.4 ± 0.2****	0.5 ± 0.0****	0.9 ± 0.0****	0.1 ± 0.0****	0.2 ± 0.0****	1.1 ± 0.1****	855	1036	1,2,3
13.759	Camphene	0.3 ± 0.0	0.3 ± 0.0	0.2 ± 0.0	0.2 ± 0.0	t****	t****	0.2 ± 0.0	871	1075	1,2,3
15.697	1-Octen-3-ol	0.2 ± 0.0	0.1 ± 0.0	0.1 ± 0.0	0.4 ± 0.0	t	t	0.2 ± 0.0	896	1451	1,2
16.625	1-Nonen-3-ol	–	–	t	–	t	0.1 ± 0.0	0.2 ± 0.0	908		1,2
17.144	β-Pinene	0.7 ± 0.0	0.4 ± 0.0*	0.3 ± 0.0**	0.4 ± 0.0****	0.1 ± 0.0****	0.3 ± 0.0**	2.1 ± 0.2°°°°	914	1110	1,2,3
17.785	α-Phellandrene	–	0.1 ± 0.0	t	t	t	t	0.3 ± 0.0	923	1160	1,2,3
18.684	δ-2-Carene	1.1 ± 0.1	0.7 ± 0.0**	t****	0.6 ± 0.1	0.1 ± 0.0****	0.5 ± 0.0****	2.0 ± 0.1°°°°	934	1146	1.2,3
19.424	*p*-Cymene	**17.7 ± 0.2**	14.1 ± 0.2****	10.2 ± 0.1****	16.9 ± 0.1****	1.6 ± 0.2****	6.1 ± 0.3****	6.5 ± 0.3****	944	1179	1,2,3
19.666	1,8-Cineole	–	–	–	–	0.2 ± 0.0	0.5 ± 0.0	1.1 ± 0.1	947	1210	1,2,3
19.982	1,3,8-*p*-Menthatriene	–	–	–	–	–	0.1 ± 0.0	–	951		1,2
20.646	β-Ocimene	–	–	–	–	t	0.2 ± 0.0	1.0 ± 0.1	960		1,2,3
21.334	Santolina triene	–	–	–	–	–	0.1 ± 0.0	–	969	1043	1,2
21.883	γ-Terpinene	**5.8 ± 0.1**	2.8 ± 0.1****	1.1 ± 0.2****	3.7 ± 0.3****	0.8 ± 0.0****	2.4 ± 0.09****	**14.2 ± 0.3**°°°°	976	1221	1,2,3
22.442	Terpinolene	–	0.4 ± 0.0	0.4 ± 0.0	–	0.2 ± 0.0	0.3 ± 0.0	–	983	1291	1,2,3
22.725	*cis*-Sabinene hydrate	–	0.2 ± 0.0	–	–	t	t	0.3 ± 0.0	1009	1470	1,2
24.669	Linalool	0.1 ± 0.0	–	0.3 ± 0.0	–	0.6 ± 0.1°°°°	0.7 ± 0.0°°°°	1.5 ± 0.2°°°°	1014	1537	1,2,3
25.096	*allo*-Ocimene	–	–	–	0.1 ± 0.0	0.1 ± 0.0	t	0.5 ± 0.1	1041	1382	1,2
27.09	diihydro–Linalool	–	t	t	–	t	t	0.5 ± 0.0	1046		1,2
29.372	Isoborneol	0.4 ± 0.0	0.9 ± 0.06°°°°	0.8 ± 0.1°°	0.5 ± 0.1	0.4 ± 0.1	0.5 ± 0.1	0.3 ± 0.0	1072	1642	1,2
30.253	Terpinen-4-ol	0.1 ± 0.0	0.1 ± 0.0	0.2 ± 0.0	0.2 ± 0.0	0.3 ± 0.0	0.4 ± 0.0°	0.5 ± 0.0°°	1083	1590	1,2,3
31.487	α-Terpineol	–	t	0.1 ± 0.0	–	0.1 ± 0.0	0.1 ± 0.0	0.2 ± 0.0	1100	1662	1,2,3
32.349	*cis*-dihydro Carvone	–	–	–	–	0.1 ± 0.0	0.1 ± 0.0	t	1106		1,2,3
34.516	Thymol, methyl ether	0.6 ± 0.0	0.4 ± 0.0	0.5 ± 0.1	0.9 ± 0.1°	1.8 ± 0.3°°°°	1.2 ± 0.1°°°°	0.9 ± 0.1°	1137	1597	1,2
35.585	Tymoquinone	–	t	**3.8 ± 0.2**	1.1 ± 0.2	0.5 ± 0.0	**3.5 ± 0.2**	0.5 ± 0.0	1152		1,2
40.059	Carvacrol	**67.8 ± 0.5**	**73.9 ± 0.6**°°°°	**76.1 ± 0.6**°°°°	**68.6 ± 0.4**°°°°	**81.4 ± 0.6**°°°°	**74.5 ± 0.4**°°°°	56.2 ± 0.3****	1211	2225	1,2,3
44.643	Carvacryl acetate	–	–	–	–	0.1 ± 0.0	0.3 ± 0.07	0.1 ± 0.0	1280	1868	1,2
46.185	Caryophyllene	1.3 ± 0.2	1.3 ± 0.2	1.5 ± 0.4	–	1.3 ± 0.0	2.2 ± 0.2	2.3 ± 0.1	1297	1575	1,2,3
46.294	Isocaryophillene	–	0.1 ± 0.0	0.1 ± 0.0	**2.4 ± 0.3**	1.4 ± 0.1	0.1 ± 0.0	1.2 ± 0.1	1299		1,2
47.157	α-Humulene	0.1 ± 0.1	–	0.1 ± 0.0	0.2 ± 0.0	0.5 ± 0.0°°	0.1 ± 0.0	t	1313	1671	1,2,3
48.297	Germacrene A	0.4 ± 0.2	0.1 ± 0.0**	0.1 ± 0.1**	0.3 ± 0.0	1.8 ± 0.1°°°°	0.8 ± 0.0*	1.4 ± 0.2°°°°	1359	1747	1,2,3
50.974	Caryophyllene oxide	–	–	0.1 ± 0.0	–	0.5 ± 0.1	0.3 ± 0.0	0.3 ± 0.0	1375	1989	1,2,3
51.985	Aromandrene	–	0.6 ± 0.1	0.7 ± 0.0	0.2 ± 0.0	1.4 ± 0.1	1.0 ± 0.1	1.7 ± 0.1	1391		1,2,3
52.398	*β*–Ylangene	–	–	0.1 ± 0.0	–	0.4 ± 0.0	0.4 ± 0.1	0.4 ± 0.0	1398	1589	1,2,3
54.056	α-Bisabolol	–	–	0.4 ± 0.0	–	1.2 ± 0.1	0.2 ± 0.0	0.2 ± 0.0	1421	2232	1,2,3
57.491	Isodaucene	–	–	–	–	t	t	–	1479		1,2
57.74	Cubebol	–	–	–	–	0.1 ± 0.0	–	–	1483	1957	1,2,3
59.449	δ-Cadinene	–	–	t	–	0.2 ± 0.0	t	–	1506		1,2,3
60.288	Germacrene B	–	–	–	–	0.1 ± 0.0	–	0.1 ± 0.0	1521	1776	1,2,3
62.008	Cycloisolongifolene, 8-hydro	–	–	–	–	0.2 ± 0.0	–	–	1552		1,2
	Total	98.8	97.9	98.0	98.0	97.8	97.7	97.9			
	Monoterpenes hydrocarbons	37.5	38.1	28.6	44.4	30.8	36.6	27.3			
	Oxygenated monoterpenes	37.5	42.9	39.3	27.8	33.3	34.1	42.4			
	Sesquiterpenes hydrocarbons	25.0	19.0	25.0	27.8	25.6	24.4	24.2			
	Oxygenated sesquiterpene			7.1		10.3	4.9	6.1			
	Yield (% w/w)	0.2 ± 0.0	0.7 ± 0.0	0.4 ± 0.0	0.1 ± 0.0	0.2 ± 0.0	2.2 ± 0.0	0.0 ± 0.0			

Significant values are in bold.

*Significantly different at p value < 0.05, **p value < 0.01; ***p value < 0.001; ****p value < 0.0001 vs. fresh; °significantly different p value < 0.05; °°p value < 0.01; °°°p value < 0.001; °°°°p value < 0.0001 vs. fresh; ^a,b^are the Kovats retention indices determined relative to a series of *n*-alkanes (C_10_–C_35_) on the apolar HP-5 MS and the polar HP Innowax capillary columns, respectively; ^c^identification method: 1 = comparison of the Kovats retention indices with published data; 2 = comparison of mass spectra with those listed in the NIST 02 and Wiley 275 libraries and with published data; 3 = coinjection with authentic compounds; c -: not detected; t = trace (< 0.1%).

**Figure 3:**
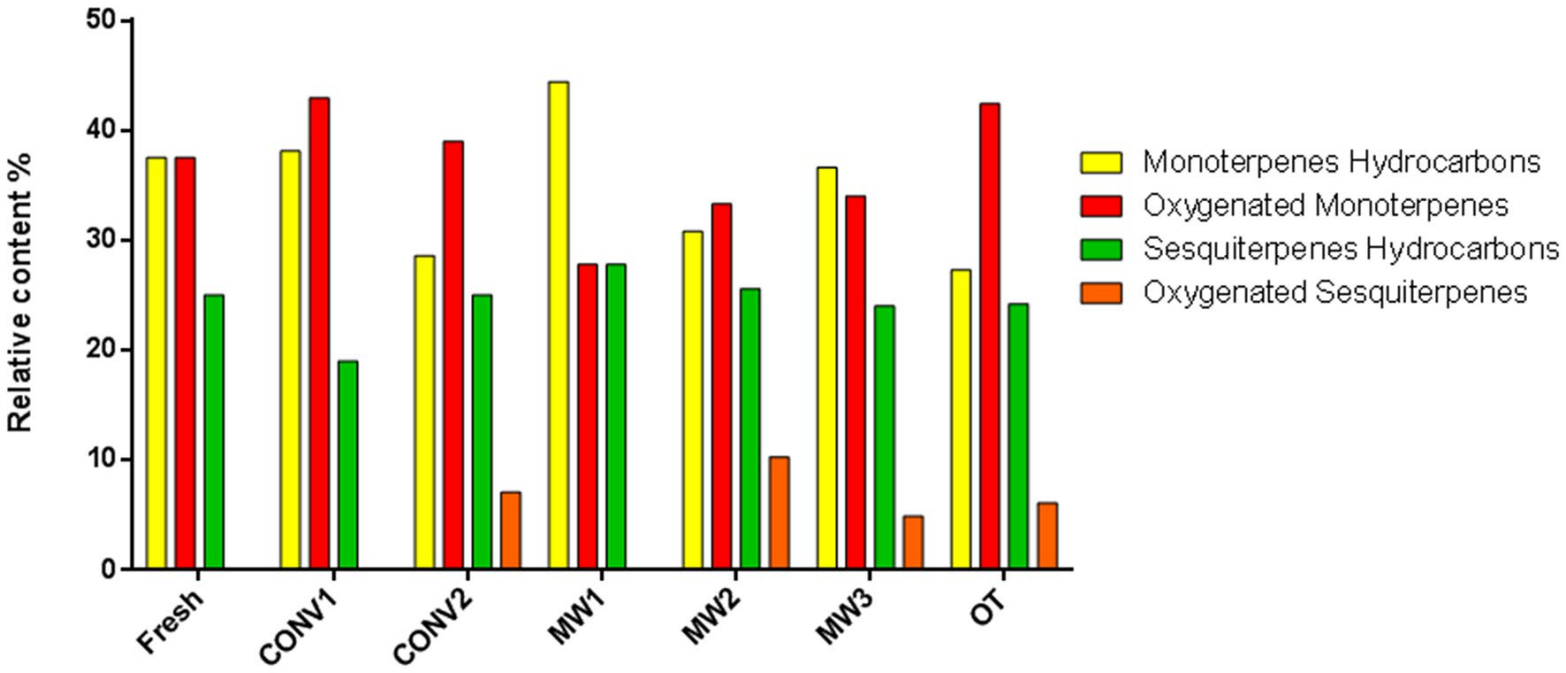
Percent composition of main chemical groups in the essential oils subjected to different treatments.

Additionally, changes in the percentages of the main chemical groups are reported in Fig. 3. Monoterpene hydrocarbons represent 27.3–44% of the total EOs, oxygenated monoterpenes represent 27.8–42.9%, sesquiterpenes represent 19–27.8% and oxygenated sesquiterpenes represent 4.9–10.3%.

A possible factor influencing the observed changes among the treatments can be related to the different molecular weights; in fact, monoterpenes have lower molecular weights than sesquiterpenes^35^ and thus could be driven from the tissue during the drying process and easily evaporated. In fact, monoterpenic hydrocarbons, which are the smallest and most volatile compounds present in essential oils, are obviously more abundant in the oil obtained from the fresh sample than in the oils obtained from the dried samples.

Oxygenated monoterpenes were mainly present in the oil obtained from samples dried by convective treatment at room conditions (CONV1). Oxygenated sesquiterpenes were present only in the essential oils from samples dehydrated by convective hot air conditions (CONV2), microwave heating (MW2 and MW3) and osmotic treatment (OT). These differences in chemical composition could be due both to a hydrolytic process and to oxidation reactions^36^. Moreover, as reported by Ghasemi Pirbalouti et al.^34^, the increase in temperature caused a conversion of some monoterpenes into sesquiterpenes.

The different drying methods used influenced the chemical composition of the EOs, with many differences in the number and percentage of constituents, as reported in Table 1. Some constituents were present in significantly greater quantities in the fresh sample than in the dried samples (highlighted with* in Table 1).

Carvacrol (ranging from 56.2 to 81.4%) was the main constituent in all samples. Specifically, the compound showed different amounts in relation to drying methods with respect to the fresh plant: in fact, carvacrol increased with all drying methods (*p* < 0.0001) except for OT, in which a significant decrease (*p* < 0.0001) in compound was compared to the fresh sample. This finding agrees with previous studies^1,37,38^.

In particular, major differences between fresh and dried samples were observed for α-pinene (0.1–2.1%), *p*-cymene (1.6–17.7%) and γ-terpinene (0.8–14.2%); in fact, these compounds were present in lower percentages in almost all dried samples than in the fresh material (*p* < 0.0001); on the other hand, γ-terpinene was present in higher amounts in samples after osmotic treatment (OT) (*p* < 0.0001) than in the fresh sample. Additionally, *β*-pinene was present in a lesser amount in all dried samples than in the fresh sample, mainly in MW1 and MW2, with a *p* value < 0.0001; the same compound was present in higher amounts in samples after osmotic treatment (OT) (*p* < 0.0001) than in the fresh sample.

**Figure 4:**
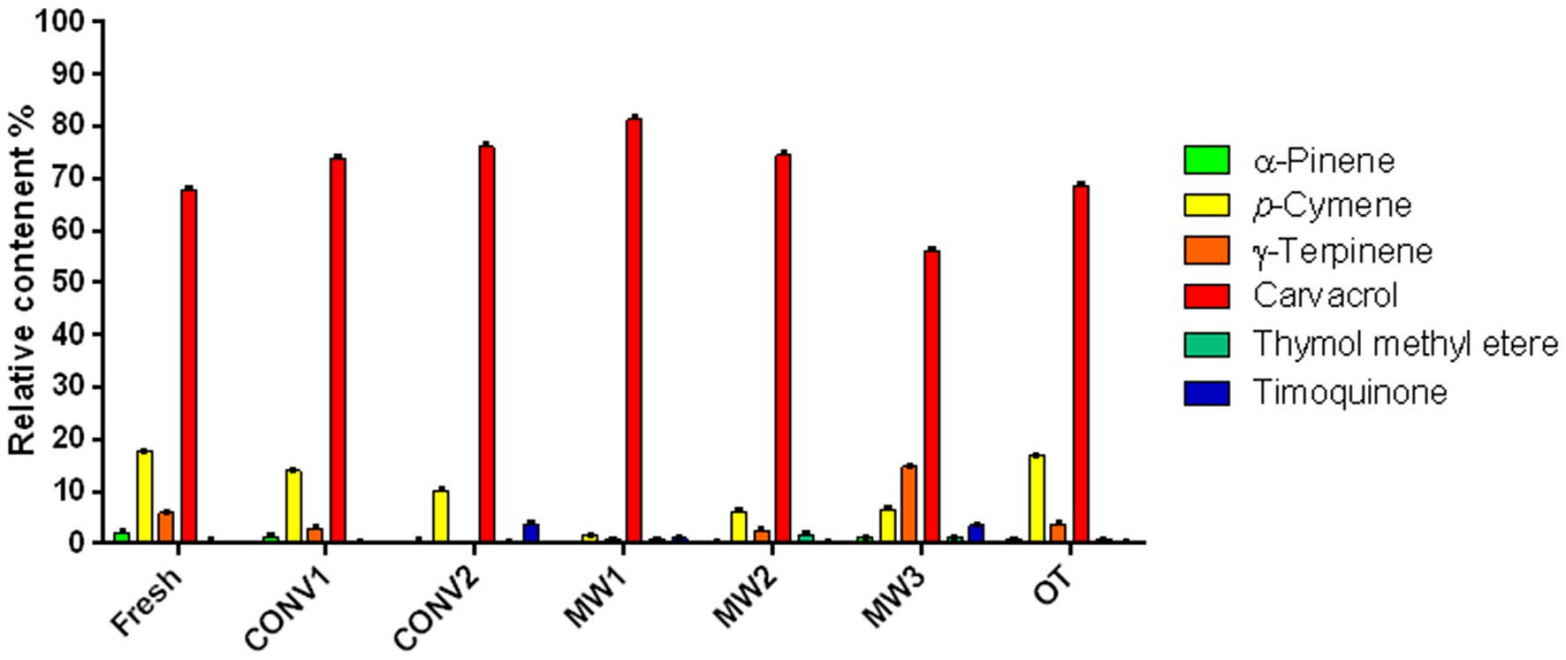
Percentage of major components of *O. vulgare* essential oils obtained by different treatments.

Thimoquinone (0.5–3.5%), thymol methyl ether (0.4–1.8%), and oxygenated monoterpenes were also present in lower amounts in fresh samples than in dried samples (Fig. 4).

Additionally, linalool, another oxygenated monoterpene, was abundant in the MW1, MW2 and OT samples (*p* < 0.0001). Isoborneol was present in higher amounts in samples obtained with shade and hot air-drying methods (CONV1 and CONV2) than in the fresh sample (*p* < 0.0001 for CONV1 and *p* < 0.01 for CONV2).

For germacrene A, an increase in the percentage of MW1 and OT samples (*p* < 0.0001) and a decrease of *p* < 0.05 for the MW3 sample and *p* < 0.01 for the CONV1 and CONV2 samples were observed.

Moreover, other constituents were not detected in some treatments, but they were present in others. For example, aromadendrene and *iso*-caryophyllene were present in all EOs except in those obtained from fresh samples; only traces of α-bisabolol were found in the EOs extracted from dried samples. In general, oxygenated sesquiterpenes, not detected in fresh samples, are present in some dried samples in percentages ranging from 4.9 to 10.3%. These results agree that the drying methods probably contribute to increasing the relative quantity of oxidized substances present. Our results corroborate other studies that showed the presence of essential oil components in dried samples that were absent in essential oil distilled from fresh material^30,31^. As previously reported, there are some studies conducted on the effect of drying on the yield and the chemical composition of essential oils extracted from Lamiaceae. Specifically, Ahmed et al.^21^ reported that the application of several drying techniques did not cause qualitative changes in the EO of *M. pulegium* with respect to oil obtained from fresh samples but slight quantitative variations in the content of their main compounds; the same changes were also registered for other Lamiaceae, including *O. basilicum*^34^ and *T. daenensis*^30^. Additionally, in our case, drying methods influenced the content of the main compounds.

## Methods

### Plant material

Aerial parts of *O. vulgare* L. (20 kg) supplied by the agricultural company Caselle of Pontecagnano, Salerno, Italy, were collected in July 2020. The species was identified by Prof. V. De Feo. A voucher specimen, labeled DF/2020/311, has been deposited in the herbarium of the Medical Botany Chair of the University of Salerno. This study complies with relevant institutional, national, and international guidelines and legislation.

### Moisture contents

Plant material (fresh and dried products) was subjected to moisture content (MC) measurements by using an Ohaus moisture analyzer (mod MB45). The working principles of the measurement method were based on thermogravimetric principles (controlled heating at a defined temperature and weighing until a constant mass value was achieved). The moisture content was expressed as the water content on a wet basis (automatic value as output of the instrument), defined as follows:

$$MC\% = \frac{wet \,material \,weight - dry \,material \,weight}{{wet \,material \,weight}} \times 100.$$

The results are reported as average values of three measurements with standard deviation (SD).

### Applied drying methods

In this study, an approach based on simplified protocols was followed in microwave heating to an easy transposition to a productive scale.

Three drying methods were applied to study the impact dehydration mechanism on the yield and chemical composition of the extracted essential oil. To this aim, the collected plant material was divided into several parts. One part was used for characterization as fresh material (control); the remaining parts were used for drying by convective methodology (shade drying—CONV1; hot-air convective drying—CONV2), by assisted microwave heating (at different operative conditions—MW 1, MW2, MW3) and by osmotic treatment (semidry technique—OT). In Table 2, sample codes and notes on selected operative conditions of applied drying methods are summarized.

**Table 2:** Samples code and note on selected operative conditions of applied drying methods.

Samples code^a^	Drying method/operative parameters
CONV1	Shade drying/shady room conditions, 5 days
CONV2	Hot-air drying/static oven at 50 °C, 24 h
MW1	Assisted microwave heating/2300 W, 10 min
MW2	Assisted microwave heating/1150 W, 15 min
MW3	Assisted microwave heating/460 W, 35 min
OT	Osmotic treatment

^a^Untreated aerial parts are indicated as fresh material.

#### Convective drying

##### Shade drying (CONV1)

The aerial parts of *O. vulgare*, covered with filter papers (aerated shady conditions), were placed on a plane surface to form a batch sample with a thickness of a few centimeters and allowed to dry at room temperature (25 ± 5 °C) for 5 days.

##### Hot-air convective drying (CONV2)

Hot-air convective drying of the oregano was carried out in a static thermostatic oven (ISCO series 9000) placing a layer of raw material of a few centimeters on a netting support. Drying was performed by setting the set-point temperature to 50 °C, and the moisture content was monitored over time until 24 h.

#### Assisted microwave heating

Microwave drying of the oregano was performed at an operative frequency of 2450 MHz by the multimodal microwave cavity LBP 210/50 Microwave Oven 2300 W, InLand, USA managed by the True-To-Power™ system to continuously vary the power supply. The oregano aerial parts were placed on a netting support until a layer (or bed) was formed. The surface temperature of the microwave-dried products was monitored with a TASI TA601B infrared thermometer.

Several irradiating tests, varying power and treatment time, were performed to explore the thermal behaviour of the oregano and thus to define several operative conditions to apply. Selected operative conditions are summarized in Table 2. After this stage, several batches of dried oregano were obtained and subjected to hydrodistillation.

#### Osmotic treatment

Semidry oregano was obtained by a technique that linked osmosis treatment to acidification. Ten kilograms of oregano aerial parts was washed by 15 s immersion in a chloride solution (100 ppm) and then rinsed and dried with a centrifuge at 700 rpm (Edy Minor Inox, Nuova Sara, Parma, Italy). The material was cut and mixed with a solution of 20% sodium chloride and 1.2% citric acid and macerated for 20 min at room temperature. The residual water was eliminated by centrifugation at 1400 rpm. The resulting vegetal material was filtered through a sieve with meshes of 3.5 mm to remove the stems and coarse parts. The concentration index is 1/4. This material was then subjected to hydrodistillation.

#### Measurement of electrolyte leakage

Electrolyte losses from oregano tissues were evaluated as mineral enrichment of aqueous medium. To this aim, conductivity measurements were performed by a Crison basic GLP31 conductivity meter. The change in conductivity was reported as the difference between the conductivity measured at time *t* and the initial conductivity of the aqueous medium (at time t_0_):

$$conductivity\, increase = conductivity \,at \,t - conductivity \,at \,t_{0}$$

In particular, 0.30 g of dried (and fresh) products were placed in 40 ml of distilled water and gently stirred. At a given time, conductivity measurements were performed (in triplicate). The results are reported as average values with standard deviation (SD) and expressed in µS cm^−1^.

### Isolation of the volatile oil

The fresh and treated aerial parts of *O. vulgare* L. were ground in a Waring blender and then subjected to hydrodistillation according to the standard procedure described in the European Pharmacopoeia^39^ until no significant increase in the volume of the collected EO was observed (3 h). The EO yield (w/v, %) was calculated according to the following equation:

$$Yield \left(\%\right)=\frac{{W}_{0 }\times 100 }{{V}_{EO}},$$

where W_*0*_ is the plant material weight distilled and V_*EO*_ is the EO volume obtained.

The volatile oils were dissolved in *n*-hexane, filtered over anhydrous sodium sulfate, and kept under N_2_ at + 4 °C in the dark until analyses.

### GC-FID analysis

Analytical gas chromatography was conducted on a Perkin–Elmer Sigma-115 gas chromatograph equipped with FID and a data handling processor, as previously reported^40^. Separation was achieved using an HP-5 MS fused-silica capillary column (30 m × 0.25 mm i.d., 0.25 μm film thickness). Column temperature: 40 °C, with 5 min initial hold, and then to 270 °C at 2 °C/min, 270 °C (20 min); injection mode splitless (1 μL of a 1:1000 *n*-hexane solution). The injector and detector temperatures were 250 °C and 290 °C, respectively. Analysis was also run by using a fused silica HP Innowax polyethyl glycol capillary column (50 m × 0.20 mm i.d., 0.25 μm film thickness). In both cases, helium was used as the carrier gas (1.0 mL/min).

### GC/MS analysis

Analyses were performed on an Agilent 6850 Ser. II apparatus, fitted with a fused silica DB-5 capillary column (30 m × 0.25 mm i.d., 0.33 μm film thickness), coupled to an Agilent Mass Selective Detector MSD 5973, as previously reported^40^; ionization energy voltage 70 eV; electron multiplier voltage energy 2000 V. Mass spectra were scanned in the range 40–500 amu, scan time 5 scans/s. Gas chromatographic conditions were as reported in the previous paragraph; transfer line temperature, 295 °C.

### Identification of the essential oil components

Most components were identified through comparison of their Kovats retention indices (Ri) [determined relative to the tR of *n*-alkanes (C_10_–C_35_)], with either those of the literature^41–44^ or with those of authentic compounds available in our laboratory. Further identification compares their mass spectra on both columns with those of authentic compounds available in our laboratories and with either those present in NIST 02 and Wiley 275 libraries^45^. The component relative concentrations were obtained by peak area normalization. No response factors were calculated.

### Statistical analysis

All tests were repeated three times. Data from each experiment were reported as the mean ± SD and statistically analyzed by two-way ANOVA followed by Dunnett’s post-hoc test at the significance level of *p* < 0.05 using GraphPad Prism 6.0 software.

## Conclusions

In this research, the yield and chemical differences of volatile oils distilled from *O. vulgare* aerial parts subjected to six different dehydration methods were studied. The obtained data revealed that the applied drying method performed as dehydration pretreatment of aerial parts has an impact on quantitative and qualitative oil features.

It was noted that a higher yield, 2.2%, was achieved by the sample subjected to microwave heating at a lower power for 35 min (MW3), although the residual moisture content (approximately 12%) can be considered only sufficiently acceptable for safe microbiological storage of dried oregano.

Focusing on four classes of constituents (39 compounds were assayed and grouped into four different classes), oxygenated sesquiterpenes were present only in the essential oils obtained from samples dehydrated by convective hot air conditions (CONV2), microwave heating (MW2 and MW3) and osmotic treatment (OT).

Moreover, carvacrol, a compound of particular interest due to its important biological activities, is the main component in all essential oils, even with different percentages (56.2–81.4%).

From a drying process point of view, the applied approach in microwave heating, based on simplified protocols, showed reduced process time (as expected due to the ability of fresh oregano to interact with microwaves), suitable to treat in short period also large amount of harvest. This aspect is relevant for industrial implementation (requirement of only basic plant components such as microwave source/heating compartment and in–out materials conveyors) and product features (extracted essential oil) are relevant too; these enhanced features, in terms of yield (MW3 sample) and detected constituents (MW3 sample), are reasonably due to secondary effects of microwave heating on oxidation, glycoside hydrolysis, and esterification reactions.

The choice of the method for obtaining the essential oil therefore appears crucial not only in relation to the higher yield but also and above all in reference to the percentage presence of components that can direct the essential oil toward an appropriate use.

## Data Availability

The data supporting the findings of this study are available within the article and its additional files.
